# The Western Medical Department

**Published:** 1863-09

**Authors:** 


### THE WESTERN MEDICAL DEPARTMENT.

In equipping for the field the immense armies which the rebellion has called forth, one of the most serious obstacles which the Government has had to contend against is the difficulty of being prepared in all emergencies with surgical and medical appliances of all kinds for the wounded, together with the thousand and one necessaries which on such occasions are indispensable, and though easy to procure for a single individual, are very difficult to be had when those stricken down by the enemy's fire are counted by thousands. To furnish such, the Medical Department of the Army, with the co-operation of the Sanitary Commissions, have made strenuous efforts, yet in many instances after the battles which have been fought during the campaigns in Virginia, especially after that of Antietam, there was almost an entire lack of requisite medical aid and of those necessaries we have alluded to, entailing consequently much suffering on the unfortunate men who were wounded in those actions; this, too, occurring in the immediate vicinity of the great cities of the East. It is, however, gratifying to know that in the Western Departments, thanks to the profound foresight, professional skill, extensive experience, and unusual administrative ability of Colonel R. C. Wood, the Assistant Surgeon-General of the United States Army, no action has been fought, since that officer assumed the duties of Chief of the Medical Department of the West, (and which for the information of our readers, we will mention comprises fourteen of the Northwestern and Southwestern States,) after which there was any want either of surgical aid, medical attendance, appliances, or comforts for the relief of those who had suffered in the engagement. At all times there has been found in the vicinity of the scene of anticipated action these important supplies, and this fact, so patent to all, and its truth so universally acknowledged, is the more remarkable when we consider the disadvantages which, from a variety of causes, the West has labored under; the difficulties of transportation through a country hostile to the forces occupying it, and the impossibility of obtaining extraneous professional aid, in districts but sparsely settled, and frequently disloyal.

Two Commanding Generals in the West, both at this time operating in the field, have accorded unbounded praise to the Medical Department of the West, not only for unceasing efforts in being ever prepared for emergencies, but also for the ample care and accommodation it has always afforded the sick as well as the wounded of our armies.

The following extracts from a letter dated June 9th, 1863, addressed by James E. Yeatman, Esq., President of the Western Sanitary Commission, on returning from a recent visit to the scene of operations, to the President of the Chamber of Commerce of St. Louis, attest the perfection, in every particular, of the arrangements made in the vicinity of Vicksburg for the relief and comfort of the wounded soldiers:

"The arrangements of the Medical Department, under the direction of Surgeon M. Mills, U. S. A., Medical Director of the Army of Gen. Grant, were most excellent, and the transportation of the sick and wounded, under Dr. A. H. Hoff, Surgeon in charge of the hospital steamer D. A. January, were more perfect than they have ever been before."

"The wounded were so well provided for by the Medical Department that it was not necessary that our steamer should be used for hospital purposes."

Nor are the above statements the only evidence which can be brought in proof of the admirable administration of the Medical Department of the West, for it has been conceded, without exception, by Governors of States, officers, civilians and others interested, that all supplies have been in great abundance; at all times on hand for emergencies in the rapid and peculiarly arduous campaigns of the war, and far beyond the wants of the army; that visitors to the scenes of action are always not only astonished at the comforts furnished, but leave quite satisfied that the soldiers are well cared for by the Government for which they are fighting.

Further testimony than this could be readily added, were it necessary, but we think sufficient has been said to show that, in spite of the many advantages enjoyed by the Eastern Department, the Medical Department of the West will not suffer by being contrasted with it, either in its administration or the professional talent it displays.—*St. Louis Republican.*

